# Prevalence and Correlates of Underweight among Women of Reproductive Age in Nepal: A Cross-Sectional Study

**DOI:** 10.3390/ijerph191811737

**Published:** 2022-09-17

**Authors:** Kritika Rana, Ritesh Chimoriya, Nabila Binte Haque, Milan K. Piya, Romila Chimoriya, Michael Ekholuenetale, Amit Arora

**Affiliations:** 1 Translational Health Research Institute, Western Sydney University, Campbelltown, NSW 2560, Australia; 2 School of Health Sciences, Western Sydney University, Penrith, NSW 2751, Australia; 3 Health Equity Laboratory, Campbelltown, NSW 2560, Australia; 4 Philanthropy Nepal (Paropakari Nepal) Research Collaboration, Auburn, NSW 2144, Australia; 5 School of Medicine, Western Sydney University, Campbelltown, NSW 2560, Australia; 6 Department of Health Systems and Populations, Macquarie University, Macquarie Park, Sydney, NSW 2109, Australia; 7 Macarthur Diabetes Endocrinology and Metabolism Service, Camden and Campbelltown Hospitals, Campbelltown, NSW 2560, Australia; 8 Department of Pediatrics, Nepal Medical College Teaching Hospital, Kathmandu 44600, Nepal; 9 Department of Epidemiology and Medical Statistics, Faculty of Public Health, College of Medicine, University of Ibadan, Ibadan 200214, Nigeria; 10 Discipline of Child and Adolescent Health, The Children’s Hospital at Westmead Clinical School, Faculty of Medicine and Health, The University of Sydney, Westmead, NSW 2145, Australia; 11 Oral Health Services, Sydney Local Health District and Sydney Dental Hospital, NSW Health, Surry Hills, NSW 2010, Australia

**Keywords:** underweight, undernutrition, women, Nepal, BMI, sociodemographic and household environmental correlates, sustainable development goals

## Abstract

This study aimed to examine the prevalence of underweight and determine the sociodemographic and household environmental correlates of underweight among women of reproductive age in Nepal. This study also compared the time trends in the prevalence of underweight with the trends in the prevalence of overweight and obesity. This cross-sectional study was a secondary data analysis of the nationally representative population-based Nepal Demographic and Health Surveys (NDHSs). Firstly, the time trends of the prevalence of underweight (body mass index (BMI) < 18.5 kg/m^2^) among women aged 15–49 years were examined at five-year intervals, from the 1996, 2001, 2006, 2011, and 2016 NDHSs (*n* = 33,507). Secondly, the sociodemographic and household environmental correlates of underweight were examined from the latest NDHS 2016 (*n* = 6165). Univariable and multivariable logistic regression analyses were performed to examine the sociodemographic and household environmental correlates of underweight. From 1996 to 2016, the prevalence of underweight decreased from 25.3% (95% confidence interval (CI) 23.8%, 26.8%) to 16.9% (95%CI 16.0%, 17.8%), while the prevalence of overweight and obesity increased from 1.6% (95%CI 1.2%, 2.1%) to 15.6% (95%CI 14.7%, 16.5%) and 0.2% (95%CI 0.1%, 0.4%) to 4.1% (95%CI 3.6%, 4.6%), respectively. Sociodemographic factors, such as age, educational status, marital status, wealth index, and religion, were independently associated with the risk of underweight. Similarly, household environmental factors, such as province of residence, ecological zone, type of toilet facility, and household possessions, including television and mobile phone, were independently associated with the risk of underweight. Despite the declining trends, the prevalence of underweight among Nepalese women remains a public health challenge. Understanding the key sociodemographic and household environmental correlates of underweight may assist in streamlining the content of health promotion campaigns to address undernutrition and potentially mitigate adverse health outcomes.

## 1. Introduction

Undernutrition and overnutrition are both major global public health challenges contributing towards increased risk of morbidity and mortality [1,2]. Globally, approximately 462 million adults are affected by underweight (body mass index (BMI) < 18.5 kg/m^2^), while 1.9 billion adults are affected by overweight or obesity (BMI ≥ 25 kg/m^2^) [3]. According to an estimation by the World Health Organization (WHO), 149 million children across the world aged under five years are affected by stunting (low height for age), 45 million by wasting (low weight for height), and 38.9 million by overweight or obesity [1]. Alarmingly, undernutrition alone is responsible for 45% of global deaths in children under five years of age [1]. Malnutrition has consistently been recognised as a major health burden in low- and middle-income countries (LMICs) [4,5], particularly in Southern Asia and sub-Saharan Africa [4,6]. In today’s context, most LMICs are affected by the double burden of malnutrition, where undernutrition (i.e., underweight, micronutrient deficiencies) and overweight or obesity manifest simultaneously [2]. South Asian countries, such as Nepal, Bangladesh, Pakistan, and India, have had historically high levels of undernutrition, yet these countries also experience a double burden of malnutrition [7,8].

Women have been found to have a higher prevalence of undernutrition compared to men across the South Asian region, and over one-third of the world’s women with anaemia live in this region [7,8,9]. One of the potential reasons for the higher prevalence of undernutrition may be linked with the social and economic disparities where men have higher priority to nutritious food compared to women [10]. A clear gender disparity is evident within the low wealth index societies of South Asian countries because of the patriarchal social structures that still prevails [11]. Moreover, women of reproductive age (15–49 years) are more susceptible to developing undernutrition [11,12], potentially due to the unmet increased nutritional requirements during successive pregnancies. Maternal undernutrition has been recognised as a key determinant of adverse health outcomes for both mothers and their offspring [13,14], including increased rates of infant and maternal morbidity and mortality [12,14,15,16,17]. Despite progresses in reducing underweight among women in South and Southeast Asian countries, the overall pooled prevalence of underweight has been estimated to be 22.9%, which is projected to decrease to 6.6% in 2030 [18]. The prevalence of underweight as projected for 2030 in South Asian countries threatens the progress towards achieving the Sustainable Development Goals (SDGs), particularly Goal 2, which includes a target of ending all forms of malnutrition by 2030 [18,19].

Nepal is a developing country in South Asia with a growing population that has doubled from 15 million in 1981 to 29 million in 2016 [20]. Despite efforts by various local, national, and international entities towards improving nutritional status of the population as a whole, nutrition among women remains one of the major health and social concerns [21]. Similar to other countries in the region, Nepalese women are often overlooked and under-prioritised, not just within the society but in many cases within their own families [22]. Although the prevalence of underweight has been declining gradually among Nepalese population attributed to several health promotion initiatives, the prevalence of underweight among Nepalese women of has remained high for a few decades [9,21]. According to the most recent Nepal Demographic and Health Survey (NDHS) 2016, an estimated 17% of women of reproductive age (15–49 years) were affected by underweight, with a BMI < 18.5 kg/m^2^ [20]. Additionally, around 12% of children were of low birth weight, and an approximate 27% of children under-five were affected by underweight, 36% by stunting, and 10% by wasting [20]. Given the profound impacts of maternal nutritional status on maternal and child health outcomes, it is essential to determine the underlying cause of underweight among women of reproductive age in Nepal.

Underweight is a multifactorial issue resulting from a complex interplay of several causes, and it is often linked with various determinants, such as health behaviours, biological factors, food environment, social environment, and household environment [9,23]. Sociodemographic factors, including age, educational status, marital status, religion, and employment status, are often closely linked with nutritional status in South Asian countries, such as Nepal [12,24,25]. On the other hand, household environmental factors, such as place of residence, cooking fuel, type of toilet facility, source of drinking water, and housing characteristics, have been shown to influence the nutritional status of women of reproductive age [13,24,26]. Determining the sociodemographic and household environmental correlates of underweight among Nepalese women of childbearing age is essential to identify the women who are at high-risk of being affected by underweight. This will also facilitate the integration of targeted interventions into nutrition programs [13], which may assist in reducing the burden of underweight and potentially mitigate any adverse maternal and child health outcomes. 

To the best of our knowledge, no studies have explored the time trends in the prevalence of underweight among women of childbearing age. A few studies were identified that have explored the nutritional status of Nepalese adults [9,17,22,27,28], yet only one study [9] was solely focused on women of reproductive age. However, most prior studies only explored limited sociodemographic correlates of underweight, and none had an exclusive focus on underweight. Therefore, the aims of this study were to examine the time trends of the prevalence of underweight and determine the sociodemographic and household environmental correlates of underweight among women of reproductive age in Nepal. This study also compared the time trends in the prevalence of underweight with the trends in the prevalence of overweight and obesity. 

## 2. Methods

### 2.1. Study Design and Data Sources

This study was a secondary data analysis of the publicly available datasets of the NDHS [29]. Firstly, the time trends of the prevalence of underweight among women of reproductive age (15–49 years) in Nepal were examined from the five-yearly (1996, 2001, 2006, 2011, and 2016) NDHSs. Secondly, the sociodemographic and household environmental correlates of underweight were examined from the latest NDHS in 2016.

The NDHS is a nationally representative population-based cross-sectional survey conducted with technical assistance from the Demographic and Health Surveys (DHS) Program [30]. The DHS Program is implemented by Inner City Fund (ICF) International and is funded by the United States Agency for International Development (USAID) [31]. The NDHSs are conducted every five years under the leadership of the Ministry of Health by New ERA Nepal [20,32,33,34,35].

### 2.2. Sampling Design

A two- or three-stage stratified cluster sampling design was employed in the NDHSs, covering all districts of Nepal, and further stratification into urban and rural areas. Using a probability proportional to size method, wards or sub-wards (i.e., subunits of municipalities) were chosen as the primary sampling units (PSUs). From each PSU, a sample enumeration area (EA) was selected. Finally, households were either chosen from the sample PSUs or the sample EAs. The sampling design and the methodology adopted for each NDHS have previously been described in detail [20,32,33,34,35].

Specifically for the 2016 NDHS, the sample was stratified and selected in two and three stages in the rural and urban areas, respectively [20]. In rural areas, wards were selected as the PSUs, and household were selected from these sample PSUs. In urban areas, wards were selected as the PSUs, followed by selection of one EA from each PSU, and then the selection of households from the sample EAs. Firstly, 383 wards were selected with probability proportional to ward size. Finally, a total of 30 households per cluster were selected with an equal probability systematic selection. Although a total of 11,473 households were selected for the sample, interviews were completed for 11,040 households [20].

### 2.3. Data Collection

Trained field staff interviewed the households using the household questionnaire to collect information on the household characteristics. Subsequently, individual interviews were conducted with woman aged 15–49 years in that household, using the woman’s questionnaire. In order to reflect the sociodemographic characteristics relevant to Nepal, an adapted version of the standard DHS woman’s questionnaire was used. Finally, anthropometric measurements were recorded using the biomarker questionnaire at participants’ homes. Using standard DHS procedures, trained female field-staff recorded the height and weight measurements [20,32,33,34,35].

### 2.4. Sample Size

The 1996, 2001, 2006, 2011, and 2016 NDHSs include a sample size of 8429, 8726, 10,793, 12,674, and 12,862 women, respectively. The 2006, 2011, and 2016 NDHSs included all women aged 15–49 years, while the 1996 and 2001 NDHSs excluded women aged 15–49 years who had never been married. Height and weight measurements were recorded only in women with children aged three years or below in the 1996 NDHS, while the 2001–2016 NDHSs collected anthropometric measurements for all interviewed women [20,32,33,34,35]. We excluded women with missing values for height and/or weight measurements and women who were pregnant at the time of the survey. A total sample of 33,507 women aged 15–49 years were extracted from the 1996 (*n* = 3420), 2001 (*n* = 7959), 2006 (*n* = 10,116), 2011 (*n* = 5847), and 2016 (*n* = 6165) NDHSs and were included in this study.

### 2.5. Outcome Variable

The primary outcome variable of this study was the prevalence of underweight. BMI was calculated by dividing the weight in kilograms by the height in meters squared (kg/m^2^). As per the World Health Organization BMI classification, BMI was categorised as follows: underweight (<18.5 kg/m^2^), normal weight (18.5 to 24.9 kg/m^2^), overweight (25 to 29.9 kg/m^2^), and obese (≥30 kg/m^2^) [36].

### 2.6. Explanatory Variables

The potential sociodemographic and household environmental correlates of underweight investigated in this study are based on the review of existing literature and available variables contained in the NDHSs datasets. The categorisation of explanatory variables based on the NDHS datasets was derived from our previous work [24].

#### 2.6.1. Sociodemographic Factors

For individual-level factors, age (15–24, 25–34, 35–49 years), educational status (no formal education, primary, secondary, higher), and employment status (not currently employed, currently employed) were considered. For family-level factors, marital status (never married, married/living with a partner, widowed/divorced/separated), number of household members (≤5, >5), wealth index (poorest, poorer, middle, richer, richest), and religion (Hindu, Buddhist, Muslim, other) were considered. The categories for the wealth index used in this study are as provided by the DHS program and as used in the NDHSs [20]. The wealth index was computed and classified into the aforementioned categories by the DHS program using the principal component analysis technique, which has been described in detail [20,24]. 

#### 2.6.2. Household Environmental Factors

Household environmental factors consist of environmental factors, household facilities, housing characteristics, and household possessions. For environmental factors, the place of residence (urban, rural), province of residence (Province 1 to Province 7), and ecological zone (Mountain, Hill, Terai) were considered. The categorisation of the province of residence was as per the categorisation used in the NDHSs [20]. For household facilities, source of drinking water (unimproved, improved), type of toilet facility (unimproved, improved), cooking fuel (solid fuel, clean fuel), and access to electricity (no, yes) were considered. For housing characteristics, the main floor, wall, and roof materials were categorised as unimproved and improved. For household possessions, refrigerator, television, mobile phone, bicycle, and motorised vehicle (motorcycle/scooter and car/truck) were considered and were classified as no and yes. Further details on the categorisations used are presented in Supplementary Table S1.

### 2.7. Statistical Analysis

For the statistical analysis, the Statistical Package for Social Sciences, Version 25 (SPSS for MacOS, SPSS Inc., Chicago, IL, USA) was used. Firstly, from the 1996, 2001, 2006, 2011, and 2016 NDHSs, the time trends of the prevalence of underweight (BMI < 18.5 kg/m^2^) among women of reproductive age in Nepal were calculated and are reported in the form of prevalence (%). The data from each survey are not matched and are not longitudinally followed up, so the prevalence reported in the study only denotes the prevalence at the cross-sectional point of the surveys. Secondly, the sociodemographic and household environmental correlates of underweight were examined from the latest NDHS in 2016. In the descriptive analyses, the characteristics of the study participants with respect to the sociodemographic and household environmental factors are reported in the form of frequency (*n*), percentages (%), and mean ± standard deviations. Univariable and multivariable logistic regression analyses were performed to examine the sociodemographic and household environmental correlates of underweight (BMI < 18.5 kg/m^2^). Variables that were significant at *p* < 0.2 in the univariable model were then included in the multivariable model. The model was then reduced using a backward stepwise procedure, while simultaneously assessing the model fitness, in order to prevent dropping of non-significant variables that may affect the model fitness. The goodness-of-fit of the model was assessed using the Hosmer–Lemeshow statistic [37]. The final model includes those variables, which when eliminated, cause a significant change in deviance (*p* < 0.05), compared with the corresponding *X*^2^ test statistic on the relevant degrees of freedom. The unadjusted crude odds ratio (COR) and adjusted odds ratio (AOR) with a 95% confidence interval (95%CI) are reported in the results.

### 2.8. Ethical Considerations

This study is a secondary data analysis of the open access datasets of the NDHSs [29]. Following registration with the DHS program and submission of an application indicating the intended use of the NDHSs datasets, permission was granted to access the datasets for the purpose of this study. The ethical approval for all NDHSs were obtained from the ethical review board of Nepal Health Research Council and human research ethics committee of ICF Macro International. The independent review boards of New ERA and ICF Macro International provided approval for the data collection procedures and tools used for the NDHSs. Prior to data collection, the DHS Program obtained informed consent from all study participants [20,32,33,34,35].

## 3. Results

### 3.1. Time Trends of the Prevalence of Underweight from 1996 to 2016

**Figure 1:**
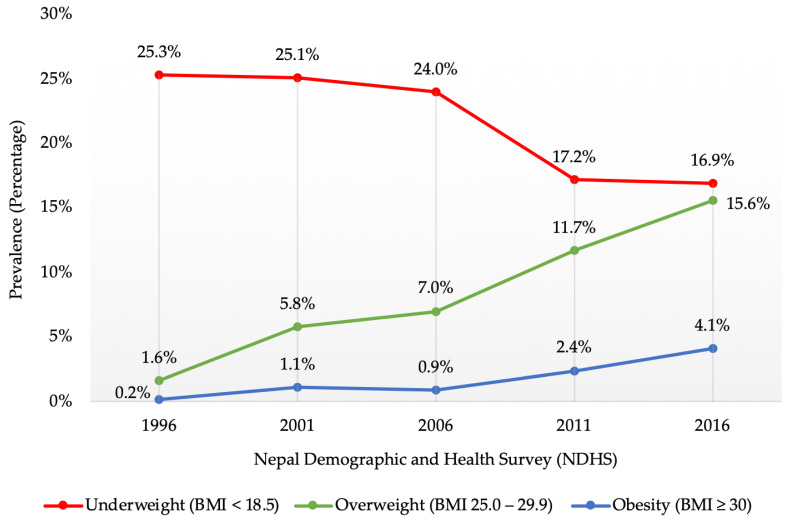
Time trends of the prevalence of underweight as compared to overweight and obesity from 1996 to 2016 among women of reproductive age in Nepal.

Figure 1 illustrates the time trends of the prevalence of underweight as compared to overweight and obesity from 1996 to 2016 among women of reproductive age in Nepal. Between 1996 and 2016, the prevalence of underweight decreased from 25.3% (95%CI 23.8%, 26.8%) to 16.9% (95%CI 16.0%, 17.8%), while the prevalence of overweight and obesity increased from 1.6% (95%CI 1.2%, 2.1%) to 15.6% (95%CI 14.7%, 16.5%) and 0.2% (95%CI 0.1%, 0.4%) to 4.1% (95%CI 3.6%, 4.6%), respectively. With the significant increase in the prevalence of overweight and obesity over the 20-year period, there has been a simultaneous decline in the prevalence of underweight. The latest NDHS 2016 illustrated that more Nepalese women were still affected by underweight compared to overweight or obesity (16.9% vs. 15.6% and 4.1%).

### 3.2. Characteristics of NDHS 2016 Study Participants

A sample of 6165 women aged 15–49 years were extracted from the latest NDHS 2016 dataset. The characteristics of the study participants in relation to the sociodemographic and household environmental factors are outlined in Table 1. The total sample had a mean weight of 50.7 ± 9.6 kg, height of 151.7 ± 5.6 cm, and BMI of 22.0 ± 3.9 kg/m^2^. Of the total sample, 16.9% were affected by underweight (*n* = 1040). 

### 3.3. Association of Sociodemographic and Household Environmental Factors with Underweight

Table 1 outlines the unadjusted association of sociodemographic and household environmental factors with underweight. In the univariable analysis, variables that were significant at *p* < 0.20 were age, educational status, employment status, marital status, number of household members, wealth index, religion, place of residence, province of residence, ecological zone, type of toilet facility, cooking fuel, access to electricity, main floor material, main wall material, and household possessions, including refrigerator, television, mobile phone, bicycle, and motorised vehicle.

Table 2 outlines the independent association of the sociodemographic and household environmental factors with underweight. In the multivariable analysis, age, educational status, marital status, wealth index, religion, province of residence, ecological zone, type of toilet facility, and household possessions, including television and mobile phone, were significantly associated with the risk of being underweight. As compared to women aged 15–24 years, those aged 35–49 years had 53% lower odds of being underweight (AOR = 0.47, 95%CI 0.37, 0.60). Women with secondary education had 27% lower likelihood of being underweight (AOR = 0.73, 95%CI 0.58, 0.93) than those with no formal education. Compared to women who were never married, those who were either widowed, divorced, or separated had 55% lower odds of being underweight (AOR = 0.45, 95%CI 0.26, 0.78). Women with the richest wealth index were 50% less likely to be affected by underweight (AOR = 0.50, 95%CI 0.35, 0.71) than those with poorest wealth index. In comparison with women who were Hindu, women who were Buddhist had 44% lower odds of being underweight (AOR = 0.56, 95%CI 0.36, 0.87). Women who resided in Province 2 had an over two-fold increase in the likelihood of being underweight (AOR = 2.04, 95%CI 1.54, 2.70) compared to women who resided in Province 1. Women living in the Terai ecological zone had 67% higher odds of being underweight (AOR =1.67, 95%CI 1.18, 2.36) than those living in the Mountain ecological zone. Compared to women with unimproved toilet facilities, those with improved toilet facilities had 30% lower odds of being underweight (AOR = 0.70, 95%CI 0.56, 0.86). Women who owned a television had 19% lower likelihood of being underweight (AOR = 0.81, 95%CI 0.67, 0.98) and women who owned a mobile phone had 37% lower odds of being underweight (AOR = 0.63, 95%CI 0.54, 0.74), compared to women who did not own a television and mobile phone, respectively.

## 4. Discussion

### 4.1. Summary of Key Findings

From the 1996, 2001, 2006, 2011, and 2016 NDHSs, the current study examined the time trends of the prevalence of underweight (<18.5 kg/m^2^) among women of reproductive age (15–49 years) in Nepal. From 1996 to 2016, the prevalence of underweight steadily decreased from 25.3% to 16.9%. However, in spite of significant increases in the prevalence of overweight and obesity, more Nepalese women were still affected by underweight than overweight or obesity (16.9% vs. 15.6% and 4.1%) in 2016. From the latest NDHS in 2016, this study examined a wide range of sociodemographic and household environmental correlates of underweight. Sociodemographic factors, such as age, educational status, marital status, wealth index, and religion, were independently associated with the risk of being underweight. Similarly, household environmental factors, such as province of residence, ecological zone, type of toilet facility, and household possessions, including television and mobile phone, were independently associated with the risk of being underweight.

### 4.2. Time Trends of the Prevalence of Underweight

The present study demonstrated a high prevalence of underweight (16.9%) among women of reproductive age in Nepal, which is consistent with the 17% presented in the NDHS 2016 report [20] and 16.8% presented in a prior study [8]. The prevalence of underweight reported in this study was higher than the rates reported for other South Asian countries, including Bangladesh (11.6%), Maldives (10.4%), and Pakistan (7.9%) [8]. These findings demonstrate that there is an urgent need to address the high prevalence of underweight among Nepalese women of reproductive age as this can lead to adverse health outcomes in women. Underweight mothers are also more likely to give birth to underweight children, causing the repetitive circle of underweight and undernutrition [15]. Moreover, being underweight may reduce a woman’s fertility while increasing her risks of adverse pregnancy outcomes, such as low birth weight, small for gestational age size, preterm birth, and neonatal death [16,17]. Undernutrition in women of reproductive age is also associated with various health complications, including pregnancy and birth related issues and uterine growth retardation leading to maternal and child mortality [12]. Therefore, there is a need to raise awareness at all levels of the society, from policymakers to families, on how maternal nutrition can have severe impacts on the development, health and survival of women and their children [12,38].

The present study identified that while there was a gradual decline in the prevalence of underweight from 1996 to 2016 (25.3% to 16.9%), there was a simultaneous gradual rise in the prevalence of overweight (1.6% to 15.6%) and obesity (0.2% to 4.1%). This finding is in line with the results for the South and Southeast Asian region, where the overall prevalence of underweight decreased from 33.2% in 1996–1999 to 21.6% in 2012–2016, whilst there was a rise in the prevalence of overweight and obesity [18]. This study also highlighted a dual burden of underweight and overweight or obesity among Nepalese women of reproductive age, which has also been confirmed by prior studies among Nepalese adults [22,27]. These findings indicate that while underweight is declining, it is still a major health burden in South Asian countries, including Nepal, and this requires development and implementation of policies and public health approaches that consider the double burden of underweight and overweight [39]. The high prevalence of underweight could be linked with the gaps in national policies, programmes, and care services particularly during pregnancy, combined with poverty and customary practices, which mean that women often fail to receive the nutritional care they need for a healthy pregnancy [40]. The lack of investment in maternal health services, the low prioritisation of nutrition services and the restricted reach of care for pregnant women further compound the difficulties [12,38]. Given the burden of women’s chronic undernutrition across generations, it is essential to prioritise women’s nutritional status, which could be the key to breaking the vicious cycle of underweight and undernutrition in Nepal [16,17].

### 4.3. Sociodemographic Factors Associated with Underweight

In the current study, sociodemographic factors, such as age, educational status, marital status, wealth index, and religion, were independently associated with the risk of being underweight. Younger women (15–24 years) in this study were more likely to be affected by underweight as compared to older women (25–34 years; 35–49 years). This is in line with the findings of prior studies conducted in Nepal and South Asian countries [39,41,42]. Similarly, women who were never married had higher odds of being underweight than women who were either married/living with a partner or widowed/divorced/separated. This is consistent with a study conducted in Bangladesh where women who were not in union had higher odds of being affected by underweight than currently married women [42]. The finding that older and married women were less likely to be affected by underweight could be linked with gestational weight gain [43], or weight gain with the use of hormonal contraceptives [44]. Therefore, interventions designed to address underweight among Nepalese women of reproductive age could be targeted towards younger and never married women who seem to be at a higher risk of being underweight.

Women with no formal education had higher odds of being underweight in this study than those with formal education (primary, secondary, or higher), which is consistent with the findings from Bangladesh [45] and South and Southeast Asian countries [18]. This finding could be linked with the manual and labour-intensive jobs that women with no formal education could be likely to engage in compared to educated women who may have sedentary occupations [24,46]. Women with the poorest wealth index were most likely to be affected by underweight, while women with the richest wealth index were least likely to be underweight. This is in line with the findings of previous studies in South Asian countries [18,39,45]. The lower odds of underweight among those with richest wealth index could be linked with the reduced likelihood of having labour-intensive jobs and increased income resulting in a greater purchasing ability and consumption of nutritious foods [24]. This study also found an association between religion and being underweight. Dietary patterns and traditional cultural beliefs may have played a vital role in this relationship [24,47]. For instance, pork and alcohol consumption is prohibited in Islam, while vegetarianism is encouraged in Hinduism, which could partly explain the study findings [24,47,48].

### 4.4. Household Environmental Factors Associated with Underweight

In the present study, household environmental factors, such as province of residence, ecological zone, type of toilet facility, and household possessions, including television and mobile phone, were independently associated with the risk of being underweight. The highest odds of having underweight was observed among women residing in Province 2 and the lowest odds among those residing in Province 4. This could be attributed to the fact that underweight is more prevalent in less affluent provinces while overweight and obesity have been found to be more prevalent in provinces with higher affluence [24,27]. For instance, Province 2 is one of the least developed provinces with human development index (HDI) of 0.422, while Province 4 is one of the most developed with HDI of 0.493 [27]. Women residing in the Terai region were more likely to be affected by underweight compared to those living in Mountain or Hill region, as found in a prior study among Nepalese adults [22]. These findings suggest that the geographical location of residence plays an essential role in the nutritional status of women of reproductive age and should be taken into consideration while developing health promotion interventions.

In this study, women with an unimproved toilet facility were more likely to be affected by underweight than those with an improved facility. An unimproved toilet facility may have a substantial impact on the nutritional status of women by predisposing them to acute respiratory infections, parasitic and helminth infections, diarrhoeal diseases, and undernutrition [13,24,49]. Therefore, continued efforts to increase access to improved toilet facilities is necessary in order to improve the nutritional status of Nepalese women [24]. In this study, women who did not own a television and mobile phone had higher odds of being underweight compared to those who did not. Corroborating the findings of this study, a prior study among Nepalese women found that women who owned a television and mobile phone were likely to have overweight or obesity [24]. However, televisions and mobile phones may be used as effective mediums to raise awareness about the dual burden of underweight and overweight among women of reproductive age in Nepal. 

### 4.5. Limitations and Strengths

There are a few limitations of this study. As a secondary analysis of previously collected data, some factors which may be associated with underweight, such as dietary habits, total energy intake, and physical activity levels could not be explored in this study due to the unavailability of these variables in the NDHS datasets. The sociodemographic and household environmental correlates of underweight investigated in this study were also limited by the available variables contained in the NDHSs datasets. We also acknowledge that the results from the stepwise procedure may be affected by random variation, as many of the predictor variables may be correlated. Therefore, the variables that were excluded from the final model may still have some association with being affected by underweight. Lastly, the data from each survey are not matched, so the prevalence reported in the study only denotes the prevalence at the cross-sectional point of the surveys. Future longitudinal studies may enhance the understanding of the causal and risk factors associated with prolonged exposure.

Nonetheless, there are several strengths of this study. Firstly, the use of standardised procedures for data collection in all NDHS, such as the use of validated questionnaires, calibrated measurement tools and trained field staff, have minimised the possibility of measurement errors and confirms the validity of the study findings. Secondly, the inclusion of large nationally representative samples of women of reproductive age across urban and rural areas in Nepal across different provinces of residence, as well as ecological zones, ensures that the study findings are generalisable. Finally, this study has been successful in comprehensively exploring a broad range of sociodemographic factors and household environmental factors associated with the risk of underweight among women of reproductive age in Nepal, which may assist in streamlining the content of health promotion interventions. Furthermore, as Nepal is accelerating its universal health coverage, as well as restructuring its health structure to a newly constructed federal structure [50], the study findings may also guide policy reforms.

## 5. Conclusions

The prevalence of underweight among Nepalese women of reproductive age steadily decreased from 25.3% to 16.9% from 1996 to 2016, yet more women were still affected by underweight than overweight or obesity in 2016. Among the investigated sociodemographic factors, women who were younger (15–24 years), without any formal education, who were never married, and with the poorest wealth index had the highest odds of being underweight. Among the explored household environmental factors, women residing in Province 2, residing in the Terai region, with an unimproved toilet facility, and who did not own a television and a mobile phone had the highest odds of being underweight. The key sociodemographic and household environmental correlates of underweight presented in this study may assist in streamlining the content of health promotion campaigns to address undernutrition. The study findings may also facilitate the integration of targeted sociodemographic and household environmental interventions into nutrition programs, which may assist in reducing the burden of underweight and potentially mitigate any adverse maternal and child health outcomes.

## Figures and Tables

**Table 1:** Characteristics of the study participants and unadjusted association of sociodemographic and household environmental factors with underweight.

Variable	Total Sample (*n* = 6165)	Underweight (BMI < 18.5)		
	*n* (%) or Mean ± SD	COR	95%CI	*p*-Value
Weight (kg)	50.7 ± 9.6			
Height (cm)	151.7 ± 5.6			
BMI (kg/m^2^)	22.0 ± 3.9			
***Sociodemographic factors***				
*Individual-level factors*				
Age (years)				
15–24	2331 (37.8%)	ref		
25–34	1834 (29.7%)	0.46	0.39–0.54	<0.001
35–49	2000 (32.4%)	0.44	0.38–0.52	<0.001
Educational status				
No formal education	2126 (34.5%)	ref		
Primary	965 (15.7%)	0.79	0.64–0.98	0.030
Secondary	2223 (36.1%)	0.99	0.85–1.15	0.860
Higher	851 (13.8%)	0.69	0.55–0.87	0.001
Employment status				
Not currently employed	2498 (40.5%)	ref		
Currently employed	3667 (59.5%)	0.77	0.68–0.88	<0.001
*Family-level factors*				
Marital status				
Never married	1323 (21.5%)	ref		
Married/living with a partner	4671 (75.7%)	0.44	0.38–0.51	<0.001
Widowed/divorced/separated	171 (2.8%)	0.33	0.20–0.55	<0.001
Number of household members				
≤ 5	3763 (61.0%)	ref		
> 5	2402 (39.0%)	1.38	1.21–1.59	<0.001
Wealth index*				
Poorest	1310 (21.2%)	ref		
Poorer	1250 (20.3%)	1.08	0.88–1.31	0.466
Middle	1251 (20.3%)	1.08	0.88–1.31	0.472
Richer	1276 (20.7%)	0.84	0.68–1.03	0.086
Richest	1078 (17.5%)	0.43	0.34–0.56	<0.001
Religion				
Hindu	5369 (87.1%)	ref		
Buddhist	296 (4.8%)	0.45	0.30–0.68	<0.001
Muslim	267 (4.3%)	1.86	1.41–2.45	<0.001
Other	233 (3.8%)	0.51	0.32–0.79	0.003
***Household environmental factors***				
*Environmental factors*				
Place of residence				
Urban	3984 (64.6%)	ref		
Rural	2181 (35.4%)	1.20	1.05–1.38	0.008
Province of residence				
Province 1	878 (14.2%)	ref		
Province 2	984 (16.0%)	2.61	2.05–3.34	<0.001
Province 3	822 (13.3%)	0.84	0.62–1.13	0.242
Province 4	783 (12.7%)	0.64	0.47–0.89	0.007
Province 5	962 (15.6%)	1.61	1.24–2.08	<0.001
Province 6	862 (14.0%)	1.32	1.01–1.73	0.045
Province 7	874 (14.2%)	1.95	1.51–2.52	<0.001
Ecological zone				
Mountain	441 (7.2%)	ref		
Hill	2823 (45.7%)	0.90	0.67–1.20	0.458
Terai	2901 (47.1%)	1.60	1.21–2.12	0.001
*Household facilities*				
Source of drinking water				
Unimproved	344 (5.6%)	ref		
Improved	5549 (90%)	1.03	0.77–1.37	0.866
Type of toilet facility				
Unimproved	747 (12.1%)	ref		
Improved	5146 (83.5%)	0.44	0.37–0.52	<0.001
Cooking fuel				
Solid fuel	4201 (68.1%)	ref		
Clean fuel	1690 (27.4%)	0.48	0.40–0.57	<0.001
Access to electricity				
No	592 (9.6%)	ref		
Yes	5301 (86.0%)	0.62	0.50–0.75	<0.001
*Housing characteristics*				
Main floor material				
Unimproved	3815 (61.9%)	ref		
Improved	2078 (33.7%)	0.53	0.45–0.62	<0.001
Main wall material				
Unimproved	3255 (52.8%)	ref		
Improved	2638 (42.8%)	0.70	0.61–0.80	<0.001
Main roof material				
Unimproved	635 (10.3%)	ref		
Improved	5258 (85.3%)	0.88	0.71–1.09	0.232
*Household possessions*				
Refrigerator				
No	5013 (81.3%)	ref		
Yes	880 (14.3%)	0.47	0.37–0.59	<0.001
Television				
No	2793 (45.3%)	ref		
Yes	3100 (50.3%)	0.57	0.50–0.66	<0.001
Mobile phone				
No	1747 (28.3%)	ref		
Yes	4418 (71.7%)	0.46	0.41–0.53	<0.001
Bicycle				
No	3522 (57.1%)	ref		
Yes	2371 (38.5%)	1.45	1.26–1.66	<0.001
Motorised vehicle				
No	4782 (77.6%)	ref		
Yes	1111 (18.0%)	0.75	0.62–0.90	0.002

The total of the categories might not always add up to the total sample due to missing data for some items. *n*: sample size. SD: standard deviation. BMI: body mass index. COR: crude odds ratio. 95%CI: 95% confidence interval. ref: reference category. * The categories for the wealth index are as provided by the DHS program.

**Table 2:** Independent association of the sociodemographic and household environmental factors with underweight.

Variable	Underweight (BMI < 18.5)		
	AOR	95%CI	*p*-Value
***Sociodemographic factors***			
*Individual-level factors*			
Age (years)			
15–24	ref		
25–34	0.57	0.46–0.71	<0.001
35–49	0.47	0.37–0.60	<0.001
Educational status			
No formal education	ref		
Primary	0.76	0.60–0.97	0.027
Secondary	0.73	0.58–0.93	0.010
Higher	0.77	0.56–1.05	0.099
*Family-level factors*			
Marital status			
Never married	ref		
Married/living with a partner	0.54	0.44–0.67	<0.001
Widowed/divorced/separated	0.45	0.26–0.78	0.005
Wealth index*			
Poorest	ref		
Poorer	0.99	0.79–1.23	0.894
Middle	0.76	0.58–0.99	0.040
Richer	0.75	0.56–1.01	0.055
Richest	0.50	0.35–0.71	<0.001
Religion			
Hindu	ref		
Buddhist	0.56	0.36–0.87	0.011
Muslim	1.08	0.78–1.48	0.655
Other	0.56	0.35–0.90	0.016
***Household environmental factors***			
*Environmental factors*			
Province of residence			
Province 1	ref		
Province 2	2.04	1.54–2.70	<0.001
Province 3	1.08	0.77–1.51	0.671
Province 4	0.82	0.58–1.17	0.266
Province 5	1.41	1.07–1.86	0.016
Province 6	1.30	0.95–1.79	0.102
Province 7	1.67	1.27–2.21	<0.001
Ecological zone			
Mountain	ref		
Hill	1.24	0.91–1.69	0.176
Terai	1.67	1.18–2.36	0.004
*Household facilities*			
Type of toilet facility			
Unimproved	ref		
Improved	0.70	0.56–0.86	<0.001
*Household possessions*			
Television			
No	ref		
Yes	0.81	0.67–0.98	0.030
Mobile phone			
No	ref		
Yes	0.63	0.54–0.74	<0.001

The final model includes those variables, which when eliminated, cause a significant change in deviance (*p* < 0.05), compared with the corresponding *X*^2^ test statistic on the relevant degrees of freedom. BMI: body mass index. AOR: adjusted odds ratio. 95%CI: 95% confidence interval. ref: reference category. * The categories for the wealth index are as provided by the DHS program.

## Data Availability

Restrictions apply to the availability of the data used in this study. The data were obtained from the DHS Program website and are available at: https://dhsprogram.com/data/available-datasets.cfm (accessed on 10 June 2022) with permission of DHS Program.

## Supplementary Materials

The following supporting information can be downloaded at: https://www.mdpi.com/article/10.3390/ijerph191811737/s1, Table S1: Explanatory variables categorisation based on the NDHS datasets.
