# Supplementary material for: Prevalence and Correlates of Underweight among Women of Reproductive Age in Nepal: A Cross-Sectional Study
Source: Int J Environ Res Public Health. 2022 Sep 17;19(18):11737. doi: 10.3390/ijerph191811737 (PMC9516984; doi:10.3390/ijerph191811737)
Supplement: Supplementary file 1 [file ijerph-19-11737-s001.zip › ijerph-1855904-supplementary.pdf]

Supplementary Materials:

Table S1. Explanatory variables categorisation based on the NDHS datasets.

Categories	Sub-Categories	Available DHS Categories
Source of drinking water	Unimproved	unprotected dug well/spring; tanker truck/cart with small tank; surface water; other
	Improved	piped into dwelling/yard/plot; piped to neighbour; public tap/standpipe; tube well or borehole; protected dug well; protected spring; rainwater; bottled water
Type of toilet facility	Unimproved	flush/pour flush not to sewer/septic tank/pit latrine; pit latrine without slab/open pit; other; no facility/bush/field
	Improved	flush/pour flush to piped sewer system/septic tank/pit latrine; ventilated improved pit (VIP) latrine; pit latrine with slab; composting toilet
Cooking fuel	Solid fuel	wood; straw/shrubs/grass; animal dung; agricultural crop; coal/ignite; charcoal; other
	Clean fuel	electricity; LPG; natural gas; biogas; kerosene
Main floor material	Unimproved	earth/sand; dung; wood planks; palm/bamboo; other
	Improved	parquet or polished wood; vinyl or asphalt strips; ceramic tiles; cement; carpet
Main wall material	Unimproved	no wall; cane/palm/trunks; mud/sand; bamboo with mud; stone with mud; plywood; cardboard; reused wood; metal/galvanized sheet; other
	Improved	cement; stone with lime/cement; bricks; cement blocks; wood planks/shingles
Main roof material	Unimproved	no roof; thatch/palm leaf; mud; rustic mat; palm/bamboo; wood planks; cardboard; other
	Improved	galvanized sheet/metal; wood; calamine/cement fibre; ceramic tiles; cement; roofing shingles

Note: The categorisation of explanatory variables based on the Nepal Demographic and Health Survey (NDHS) datasets was derived from our previous work [24].
